# Cardiometabolic Health in Submariners Returning from a 3-Month Patrol

**DOI:** 10.3390/nu8020085

**Published:** 2016-02-09

**Authors:** Heath G. Gasier, Colin R. Young, Erin Gaffney-Stomberg, Douglas C. McAdams, Laura J. Lutz, James P. McClung

**Affiliations:** 1 Department of Military and Emergency Medicine, Uniformed Services University of the Health Sciences, Bethesda, MD 20814, USA; 2 Naval Submarine Medical Research Laboratory, Groton, CT 06349, USA; colin.r.young.mil@mail.mil; 3 United States Army Research Institute of Environmental Medicine, Natick, MA 01760, USA; erin.g.stomberg.civ@mail.mil (E.G.-S.); laura.j.lutz2.civ@mail.mil (L.J.L.); james.p.mcclung8.civ@mail.mil (J.P.M.); 4 Commander, Submarine Group Nine, Silverdale, WA 98315, USA; douglas.c.mcadams.mil@mail.mil

**Keywords:** adipokines, chemokines, diet, inflammation, insulin resistance, metabolic syndrome, physical activity, obesity

## Abstract

Confined space, limited exercise equipment, rotating shift work and reduced sleep may affect cardiometabolic health in submariners. To test this hypothesis, 53 male U.S. Submariners (20–39 years) were studied before and after a 3-month routine submarine patrol. Measures included anthropometrics, dietary and physical activity, biomarkers of cardiometabolic health, energy and appetite regulation, and inflammation. Before deployment, 62% of submariners had a body fat % (BF%) ≥ 25% (obesity), and of this group, 30% met the criteria for metabolic syndrome. In obese volunteers, insulin, the homeostatic model assessment of insulin resistance (HOMA-IR), leptin, the leptin/adiponectin ratio, and pro-inflammatory chemokines growth-related oncogene and macrophage-derived chemokine were significantly higher compared to non-obese submariners. Following the patrol, a significant mean reduction in body mass (5%) and fat-mass (11%) occurred in the obese group as a result of reduced energy intake (~2000 kJ) during the patrol; and, independent of group, modest improvements in serum lipids and a mean reduction in interferon γ-induced protein 10 and monocyte chemotactic protein 1 were observed. Since 43% of the submariners remained obese, and 18% continued to meet the criteria for metabolic syndrome following the patrol, the magnitude of weight loss was insufficient to completely abolish metabolic dysfunction. Submergence up to 3-months, however, does not appear to be the cause of obesity, which is similar to that of the general population.

## 1. Introduction

The estimated prevalence of overweight and obesity in U.S. adults is ~70% and 35%, respectively, rates that have more than doubled since 1976–1980 [1]. In U.S. active duty servicemembers, 60.5% are classified as overweight or obese and 12.9% as obese, a 2.5 fold increase from 1990 [2]. While the potential consequences of obesity are documented and include an increased risk of developing dyslipidemia, hypertension, metabolic syndrome, type 2 diabetes mellitus (T2DM), cardiovascular disease (CVD), and certain types of cancer [3], military readiness may also be impacted. The annual medical costs related to obesity have soared, *i.e.*, $147 billion for the general population, nearly $70 billion more than in 1998 [4], and $1.1 billion for enrollees of the Tricare Prime health plan [5]. While the reasons for the rise in obesity are, perhaps, similar between the U.S. general population and the military, the stress of military duty may present additional challenges for maintaining a healthy body weight, such as frequent deployments and relocations [6]. Also, there are certain military occupations that require residence in unique environments that may affect servicemembers ability to maintain optimal health and fitness.

One such occupation within the Navy is submarine duty. During submergence, crewmembers reside in a confined space void of sunlight, have limited access to exercise equipment, and may perform rotating shift work (6-h on duty, 12-h off duty) which causes circadian desynchrony [7]. Circadian rhythm disruption adversely affects the metabolic responses to feeding (partly due to insulin resistance), alters leptin secretion patterns favoring energy intake, and leads to dysregulated innate immunity and systemic inflammation [8,9,10,11,12]. These events contribute to the increased risk for metabolic syndrome and CVD reported in shift workers [12,13]. Thus, it is plausible that if energy intake is not offset by energy expenditure during submergence as a result of increased intake and/or a decrease in physical activity, combined with occupational demands, submariners may be at an elevated risk for obesity and metabolic syndrome.

In the present study we tested the hypothesis that the proportion of submariners classified as overweight and obese would increase following a routine 3-month patrol. In contrast to this expectation, we observed a significant mean decrease in submariner body mass and fat mass following the patrol [14]. This observation led us to explore anthropometric changes by body fat classification, non-obese and obese, using a body fat % (BF%) cutoff of 25%. Diet, physical activity, blood pressure, and serum measures of cardiometabolic health (*i.e.*, glucose, insulin, adiponectin, lipids and inflammatory cytokines) and appetite and energy homeostasis (ghrelin and leptin) were assessed.

## 2. Experimental Section

### 2.1. Subjects

Fifty-three active duty male submariners (20–39 years) assigned to a U.S. Navy submerged ship ballistic nuclear (SSBN) missile submarine at Kitsap Naval Base, Bangor, WA preparing to depart on a routine patrol volunteered to participate in this investigation as previously described [14]. The study was approved by the Navy Submarine Medical Research Laboratory’s (NSMRL) Institutional Review Board (protocol NSMRL2012.0001) in compliance with all applicable federal regulations governing the protection of human subjects.

Within 1-week of departure and return from the submarine patrol, subjects underwent testing to include anthropometrics, blood pressure and pulse, fasting blood collection, and completed food and activity frequency questionnaires.

### 2.2. Anthropometrics

Body composition, fat and fat-free mass, was determined by air displacement plethysmography (BOD POD^®^ Gold Standard System, Cosmed, Concord, CA, U.S.) [15]. The coefficients of variation for fat and fat-free mass were 1.95% and 0.52%, respectively. Wearing only Lycra shorts, height was determined with a stadiometer (Creative Health Products, Plymouth, MI, U.S.) to the nearest 0.1 cm and body mass was measured to the nearest 0.01 kg by a calibrated electronic scale attached to the BOD POD^®^. Waist circumference was determined just above the iliac crest with a tape measure to the nearest 0.1 cm. A calculated body mass index (BMI) (kg/m^2^) was used to classify submariners as normal-weight (18.5–24.9), overweight (25–29.9), or obese (≥30) [16], whereas a measured BF% of ≥25% was used to objectively determine obesity, a cutoff that has been suggested to define obesity [17,18,19,20,21].

### 2.3. Dietary Intake

A food frequency questionnaire (FFQ) was administered by Registered Dietitians (Fred Hutchinson Cancer Research Center, Seattle, WA, USA) to assess mean dietary intake in port and during deployment. The Fred Hutchinson FFQ, which has been validated in comparison to a 3-day diet record [22], lists 125 different food and beverages, and instructs the volunteers to report the portion sizes (e.g., teaspoons, cups, slices) of the foods and beverages they consumed within the previous month. In order to quantify the mean daily consumption of energy, macronutrient intake (carbohydrates, protein, fats), cholesterol, sodium, fiber, fruits and vegetable servings, and alcohol intake, questionnaires were scanned and calculated by the Nutrition Assessment Shared Resources (NASR; Fred Hutchinson Cancer Research Center) using Nutrition Data Systems for Research (NDSR) software (a U.S. Department of Agriculture Nutrient Database). Subjects with implausible reported energy intakes (*n* = 6, *i.e.*, <3347 kJ/day or >20,920 kJ/day) were excluded from analysis [23].

### 2.4. Physical Activity

An activity frequency questionnaire (AFQ) (The University of Arizona Cancer Center, Tucson, AZ, USA) was used to assess mean in port and deployment physical activity energy expenditure (PAEE). The Arizona AFQ is a 59-item questionnaire that groups physical activity by occupation, sleep, recreation, leisure, household, and personal care. PAEE was calculated by using the Compendium of Physical Activity codes, which are assigned a metabolic equivalent (MET) value, defined as the ratio of the work metabolic rate to a standard resting metabolic rate, *i.e.*, 4.184 kJ/kg/h, 1 MET [24]. Since some participants may have overestimated their daily physical activity, those who reported ≥32 h of activity per day (*n* = 16) were excluded as previously suggested [25].

### 2.5. Blood Collection and Biochemical Assessment

Under fasting conditions (minimum of 8-h), blood was collected from the antecubital vein, allowed to clot, and centrifuged at 3600 rpm for 10-min for serum separation. At the end of each testing session, frozen serum was shipped to the Pennington Biomedical Research Center (PBRC, Baton Rouge, LA, USA) and stored at −80°C until both pre- and post-patrol samples were available for batch analysis. The PBRC is accredited by the College of American Pathologists and, therefore, regularly participates in inter-lab assay validation. Serum lipids (total cholesterol, low-density lipoprotein (LDL-C) and high-density lipoprotein (HDL-C) cholesterol, and triglycerides) and glucose were determined by the oxygen electrode method using a Beckman Coulter DXC 600 Pro system (Beckman Coulter, Fullerton, CA, USA). Serum insulin was determined by immunoassay and chemiluminescence detection (Immunolite 2000, Siemens Healthcare Diagnostic’s, Inc., Tarrytown, NY, USA). Serum adiponectin, leptin and ghrelin were determined using radioimmunoassays (Millipore, Billerica, MA, USA) with a PerkinElmer Wizard 2470 gamma counter (PerkinElmer, Waltham, MA, USA). A MILLIPLEX^®^ MAP 42 human cytokine/chemokine magnetic bead panel was determined by using a Luminex^®^ fluorescence imager (Millipore, Billerica, MA, USA). Of these, 23 were determined to be above the minimum detectable concentrations and were included in analysis: epidermal growth factor, eotaxin, fibroblast growth factor 2, fractalkine, granulocyte-colony stimulating factor, granulocyte-macrophage colony-stimulating factor, growth-regulated protein (GRO), interferon α2 and γ, interferon γ-induced protein 10 (IP-10), interleukin-1 receptor antagonist, interleukins 8 and 10, interleukin-12p40, monocyte chemotactic protein 1 (MCP-1), macrophage-derived chemokine (MDC), macrophage inflammatory protein-1α and 1β, platelet-derived growth factor-AA and AB/BB, regulated on activation, normal T cell expressed and secreted (RANTES), tumor necrosis factor α and vascular endothelial growth factor.

As an index of insulin resistance, the homeostatic model assessment of insulin resistance (HOMA-IR) was calculated as previously described [26].

### 2.6. Metabolic Syndrome Assessment

Volunteers were classified as having metabolic syndrome if they had ≥ 3 of the following: a waist circumference ≥ 102 cm, triglycerides ≥ 150 mg/dL, HDL-C < 40 mg/dL, systolic blood pressure ≥ 130 mmHg, diastolic blood pressure ≥ 85 mmHg, and fasting plasma glucose ≥ 100 mg/dL [27]. By using the National Heart, Lung and Blood Institute 2005 guidelines [27], we feel that a better qualitative comparison between our sample and the U.S. general population could be made.

### 2.7. Statistical Analysis

All analyses were conducted using SigmaPlot 10.0 (Systat Software, San Jose, CA, USA). The Pearson product-moment correlation coefficient was used to determine the linear association between BF% and BMI. Chi-square tests were used to determine whether the proportion of submariners classified as normal, overweight or obese changed over the course of the patrol. A Fisher exact test was used to determine if the proportion of submariners who met the criteria for metabolic syndrome differed between non-obese and obese submariners prior to and following the patrol. A two-way repeated measures ANOVA was used to determine whether non-obese (BF% < 25%) and obese (BF% ≥ 25%) submariners responded differently to the patrol. When a significant main effect was observed, post hoc testing was completed using the Student-Newman-Keuls method. In the event that the data failed normality (Kolmogorov-Smirnov) or equal variance testing, data were transformed (log or square root) and analyzed. Since our main outcome was obesity classification, the sample size (*n* = 20, non-obese and *n* = 33, obese) yielded a power of 0.938 with an alpha of 0.05 (group × time). Data are presented as means ± SD. Values of *p* < 0.05 were considered statistically significant.

## 3. Results

### 3.1. Anthropometrics

**Figure 1:**
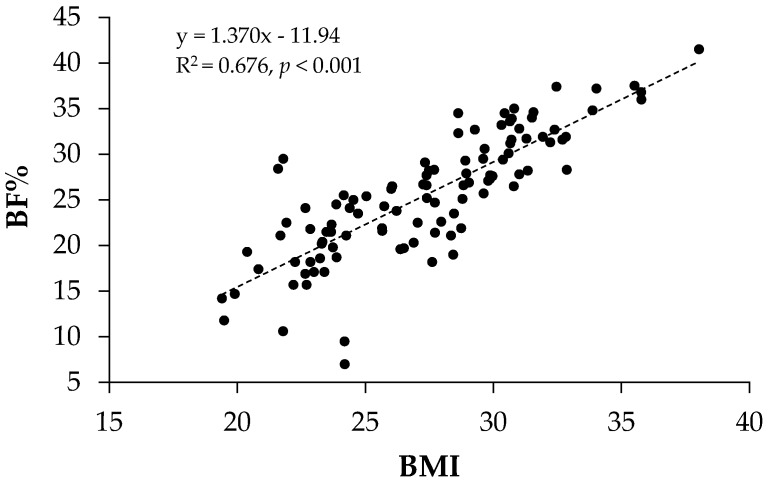
The relationship between body fat % (BF%) and body mass index (BMI) in submariners. BF% and BMI were significantly correlated, *r* = 0.822, *p* < 0.001 (*n* = 106).

Prior to deployment, the mean BMI and BF% were 27.8 ± 4.2 kg/m^2^ and 27.0 ± 6.6, respectively. When calculating BMI, 68% of the volunteers were either overweight or obese before, and 66% following the patrol; however, there was a trend for a decrease in the proportion of obese volunteers returning to port (*p* = 0.081) (Table 1). Although there was correlation between BF% measured by air displacement plethysmography and BMI (r = 0.822, *p* < 0.001, Figure 1), 29% and 44% of the volunteers were incorrectly classified as being overweight by BMI when their BF% was < 25% before and following the patrol, respectively; thus, a BF% cutoff of 25% was used in subsequent analyses (Table 2 and Table 3). By grouping the volunteers as non-obese and obese, the reduction in body mass, BMI, waist circumference and fat mass only occurred in the obese group following the patrol. As expected, fat-free mass was significantly greater in the obese group.

**Table 1:** Mean BF% according to BMI classification in submariners before and after a 3-month patrol.

Time	BMI Classification		
	Normal (18.5–24.9 kg/m^2^)	Overweight (25–29.9 kg/m^2^)	Obese (≥30 kg/m^2^)
*Pre-*	20.5 ± 4.6 (32)	26.5 ± 3.8 (32)	33.3 ± 3.4 (36)
*Post-*	18.2 ± 5.3 (34)	24.8 ± 3.7 (47)	32.4 ± 3.5 (19)

Values are means ± SD with the percentage of volunteers within each classification in parentheses. BF%, body fat %; BMI, body mass index.

### 3.2. Clinical and Health Related Characteristics

Although not significant, there was a trend towards an increased systolic blood pressure and heart rate in the obese group compared to the non-obese volunteers, and a decrease in diastolic blood pressure following the patrol independent of group (Table 2).

There was a significant group × time interaction in reported energy intake, such that the obese volunteers consumed significantly more energy while in port prior to the patrol (mean 2117 kJ). During deployment the obese volunteers decreased their mean energy intake by 2047 kJ. A substantial proportion of this reduction was attributed to a decrease in carbohydrate intake, *i.e.*, −56%. Independent of group, submariners reported consuming significantly less fruits and vegetables, fiber and alcohol during the patrol.

On average, obese volunteers slept 1 h less than non-obese volunteers, and an overall reduction in sleep of 1 h during the patrol was observed in both groups. PAEE was significantly greater in obese volunteers with no changes occurring during the patrol.

### 3.3. Biomarkers of Glucose Metabolism, Lipid Profiles, Appetite and Energy Homeostasis and Inflammation

The concentration of insulin and the calculated HOMA-IR were significantly higher in the obese volunteers (Table 3). Consistent with these data, there was a trend for a decrease in adiponectin in the obese group. Serum triglycerides and the ratio of cholesterol/HDL-C were significantly higher in the obese volunteers. Independent of group, there was an overall significant mean decrease in total cholesterol, LDL-C and the ratio of total cholesterol/HDL-C.

Leptin levels were significantly higher in the obese volunteers, and although there was a decrease in this group following the patrol, the concentration remained considerably higher than the non-obese group (Table 3). As a result of the increased leptin concentrations and a trend for a reduction in adiponectin within the obese group, the leptin/adiponectin ratio was ~3.6 fold greater in the obese compared to the non-obese volunteers. No significant differences were observed for ghrelin, however, there was a trend towards a higher level in the non-obese volunteers, and an increase following the patrol independent of group.

Of the 23 cytokines/chemokines analyzed, statistically significant differences were identified for GRO, IP-10, MCP-1 and MDC, and a trend was observed for RANTES, thus data are only shown for these 5. Overall, GRO and MDC were significantly greater (26 and 18%, respectively) in the obese group compared to the non-obese group, while a trend towards an increase in RANTES was observed in the obese submariners. Independent of group, submariners displayed a significant decrease in IP-10 (−11%) and MCP-1 (−12%) following the patrol.

### 3.4. Metabolic Syndrome

None of the non-obese volunteers met the criteria for metabolic syndrome prior to or following the patrol (data not shown). In contrast, 30% of the obese sailors had metabolic syndrome prior to departure, significantly different from the non-obese group (*p* = 0.008). The proportion of obese sailors that returned to port decreased by 12%, however, this was not significant.

**Table 2:** Anthropometrics, clinical and health related characteristics in non-obese and obese submariners before and after a 3-month patrol.

Variable	BF < 25% (*n* = 20)		BF ≥ 25% (*n* = 33)		*p* Value		
	*Pre-*	*Post-*	*Pre-*	*Post-*	*Group*	*Time*	*Int.*
*Anthropometrics*							
Height, cm	178 ± 8	-	178 ± 6	-			
Weight, kg	75 ± 8 ^a^	75 ± 9 ^b^	96 ± 12 ^a,c^	91 ± 11 ^b,c^	<0.001	<0.001	<0.001
BMI, kg/m^2^	24 ± 2 ^a^	24 ± 3 ^b^	30 ± 3 ^a,c^	29 ± 3 ^b,c^	<0.001	<0.001	<0.001
Waist circumference, cm	86 ± 6 ^a^	86 ± 6 ^b^	102 ± 6 ^a,c^	97 ± 8 ^b,c^	<0.001	<0.001	<0.001
Fat mass, kg	17 ± 5 ^a^	16 ± 6 ^b^	30 ± 5 ^a,c^	27 ± 6 ^b,c^	<0.001	<0.001	<0.001
BF%	20 ± 4 ^a^	19 ± 5 ^b^	31 ± 4 ^a,c^	27 ± 5 ^b,c^	<0.001	<0.001	<0.001
Fat-free mass, kg	60 ± 7	61 ± 7	65 ± 7	65 ± 7	0.021	0.067	0.183
*Blood pressure and pulse*							
Systolic, mmHg	126 ± 8	129 ± 15	133 ± 12	133 ± 12	0.082	0.348	0.500
Diastolic, mmHg	80 ± 5	78 ± 9	82 ± 8	80 ± 9	0.192	0.059	0.856
Heart rate, beats/min	66 ± 8	65 ± 11	72 ± 11	69 ± 21	0.094	0.603	0.695
*Daily dietary intake*							
Energy, kJ	9556 ± 2824 ^a^	9881 ± 3813	11673 ± 3935 ^a,c^	9624 ± 3766 ^c^	0.309	0.074	0.017
Carbohydrate, g	254 ± 77	260 ± 111	310 ± 121 ^c^	235 ± 111 ^c^	0.515	0.029	0.012
Protein, g	98 ± 34	105 ± 45	117 ± 42	109 ± 44	0.268	0.895	0.210
Fat, g	91 ± 28	104 ± 38	107 ± 39	106 ± 45	0.316	0.362	0.210
Saturated fat, g	29 ± 9	35 ± 13	37 ± 14	36 ± 17	0.215	0.243	0.149
Cholesterol, mg	380 ± 159	408 ± 149	441 ± 206	450 ± 261	0.303	0.624	0.769
Sodium, mg	3785 ± 1239	4049 ± 1498	4269 ± 1473	4056 ± 1385	0.455	0.995	0.247
Fruits & vegetables, servings	3 ± 1	2 ± 2	4 ± 3	2 ± 2	0.463	0.011	0.323
Fiber, g	20 ± 7	19 ± 7	23 ± 9	19 ± 6	0.439	0.045	0.086
Alcohol, servings	1 ± 1	0	2 ± 3	0	0.386	0.001	0.386
*Activity and sleep*							
Daily PAEE, kJ	3989 ± 2262	3900 ± 1989	5860 ± 2252	4794 ± 2436	0.028	0.216	0.295
Daily sleep, h/day	7 ± 1	6 ± 2	6 ± 2	5 ± 2	0.012	0.001	0.343

Values are means ± SD. PAEE, physical activity energy expenditure; *n* = 47 for dietary intake, 37 for PAEE, and 50–53 for all others. Dietary intake was calculated from food frequency questionnaire responses using the U.S. Department of Agriculture’s Database. PAEE was calculated from activity frequency questionnaire responses and assigning metabolic equivalent values for activities (occupational, sleep, recreation, leisure, household, and personal care). For significant interactions, values (^a,b,c^) sharing similar superscripts are significantly different.

**Table 3:** Biomarkers of glucose metabolism, lipid profiles, appetite and energy homeostasis and inflammation in non-obese and obese submariners before and after a 3-month patrol.

Variable	BF < 25% (*n* = 20)		BF ≥ 25% (*n* = 33)		*p* Value		
	*Pre-*	*Post-*	*Pre-*	*Post-*	*Group*	*Time*	*Int.*
Glucose, mg/dL	84 ± 6	88 ± 5	89 ± 8	89 ± 7	0.064	0.157	0.081
Insulin, mU/L	4.6 ± 1.9	5.2 ± 2.4	9.4 ± 5.0	9.8 ± 5.9	<0.001	0.430	0.747
HOMA-IR	1.0 ± 0.4	1.1 ± 0.5	2.1 ± 1.2	2.2 ± 1.4	<0.001	0.349	0.484
Adiponectin, µg/mL	6.3 ± 2.6	5.7 ± 2.6	5.0 ± 3.0	4.4 ± 2.4	0.066	0.075	0.085
Total cholesterol, mg/dL	180 ± 41	170 ± 37	184 ± 27	174 ± 29	0.644	<0.001	0.951
Triglycerides	89 ± 43	75 ± 21	162 ± 120	131 ± 76	0.001	0.098	0.612
LDL-C, mg/dL	114 ± 34	106 ± 33	112 ± 24	103 ± 29	0.804	0.003	0.706
HDL-C, mg/dL	49 ± 10	48 ± 9	44 ± 9	44 ± 8	0.061	0.745	0.785
Total cholesterol/HDL-C	3.8 ± 0.9	3.6 ± 0.8	4.4 ± 1.3	4.1 ± 1.0	0.048	0.001	0.396
Ghrelin, pg/mL	985 ± 335	1042 ± 273	848 ± 241	915 ± 277	0.065	0.053	0.795
Leptin, ng/mL	3.9 ± 2.1 ^a^	4.2 ± 2.9 ^b^	11.7 ± 5.9 ^a,c^	8.7 ± 5.1 ^b,c^	<0.001	0.100	0.017
Leptin/Adiponectin	0.8 ± 0.6	0.9 ± 0.7	3.4 ± 2.7	2.6 ± 2.2	<0.001	0.461	0.246
GRO, pg/mL	427 ± 154	429 ± 153	541 ± 221	539 ± 208	0.038	0.964	0.959
IP-10, pg/mL	311 ± 178	292 ± 156	298 ± 96	249 ± 83	0.412	0.007	0.228
MCP-1, pg/mL	554 ± 191	471 ± 197	623 ± 274	556 ± 281	0.263	0.022	0.692
MDC, pg/mL	929 ± 241	931 ± 315	1079 ± 319	1136 ± 335	0.044	0.235	0.251
RANTES, ng/mL	208 ± 127	188 ± 99	284 ± 164	248 ± 172	0.086	0.182	0.727

Values are means ± SD. HOMA-IR, homeostatic model assessment of insulin resistance; LDL-C, low-density lipoprotein; HDL-C, high-density lipoprotein; GRO, growth-related oncogene; IP-10, interferon γ-induced protein 10; MCP-1, monocyte chemotactic protein 1; MDC, macrophage-derived chemokine; RANTES, regulated on activation, normal T cell expressed and secreted. For significant interactions, values (^a,b,c^) sharing similar superscripts are significantly different.

## 4. Discussion

The primary objective of this observational study was to assess the prevalence of overweight and obesity in a cohort of submariners, and whether 3-months of submergence altered these rates. A secondary objective was to assess differences in anthropometric changes following a patrol between non-obese and obese submariners, and to determine whether these changes were accompanied by alterations in diet, physical activity, indices of cardiometabolic health, and appetite and energy regulation. The primary findings from this work are twofold: First, 62% of crewmembers were obese prior to the patrol; and the cardiometabolic health profile of obese sailors was poor compared to the non-obese sailors, as 30% of this group met the criteria for metabolic syndrome. Secondarily, contrary to our hypothesis and in spite of the occupational demands associated with prolonged submergence, submariners experienced a modest reduction in weight and improvement in some aspects of cardiometabolic health, *i.e.*, serum lipids and inflammation (IP-10 and MCP-1). However, measures including serum insulin and HOMA-IR, the leptin/adiponectin ratio, and the chemokines GRO and MDC were unchanged in the obese group following the patrol, indicating that a further reduction in body mass is likely required to correct metabolic dysfunction.

The estimated prevalence of overweight and obesity in U.S. Submariners has been reported to be ~50% and ~17.5%, respectfully, 5% higher than sailors assigned to aircraft carriers, and similar to the general population [28]. These data were derived from BMI, thus the potential for misclassification exists, *i.e.*, categorizing individuals as overweight when they have increased fat-free mass. In order to explore this possibility, a subsequent cross-sectional examination in submariners was performed comparing BMI and BF% (measured by dual-energy X-ray absorptiometry, DXA) [29]. The results revealed that, although there was correlation between BMI and BF% (Pearson’s r = 0.874), 37% of 20–39 year old male submariners were misclassified as not being obese by BMI when they were by using a BF% cut-off of 25%, indicating that the increase in BMI was not due to an increase in fat-free tissue. In the present investigation whereby air displacement plethysmography was used to assess fat and fat-free mass in a cohort of submariners residing in a different geographical location, a good correlation between BMI and BF% was also determined, with more than a third of the crew being classified as obese by BMI and nearly two-thirds by BF%. Taken together, these data confirm that the prevalence of obesity in submariners is high and similar to the general population [21,30,31].

Although most of the mean clinical and laboratory data were within normal ranges within the young (29 ± 5 years) group of submariners studied, the consequences of obesity were already apparent. Specifically, nearly one-third of the obese group had metabolic syndrome, which was 19% of the crewmembers studied and similar to the prevalence in 20–39 year old males of the U.S. general population (~21%) [32]. Also in the obese group, 30% were insulin resistant using a HOMA-IR cutoff of ≥ 2.73 [33], and adiponectin, which is an adipokine that has insulin-sensitizing activity, trended toward a reduction in obese submariners, a finding previously reported in adults with metabolic syndrome, insulin resistance and T2DM [34]. The underlying mechanism for the obesity associated onset of insulin resistance and progression to T2DM has been proposed to be due to chronic low-grade inflammation [35]. In the present study, the obese volunteers displayed a mean increase in GRO and MDC, and a trend towards an increase in RANTES, all possessing pro-inflammatory properties. GRO and RANTES have previously been reported to be increased in obese adults [36,37], while to our knowledge, this is the first description of MDC being increased in young obese males. In addition, leptin, an adipokine that suppresses appetite, increases energy expenditure and promotes weight loss was nearly double in the obese submariners, indicating the development of leptin resistance [38,39]. The increased leptin/adiponectin ratio in the obese volunteers suggests an imbalance between pro- (leptin) and anti-inflammatory (adiponectin) control, favoring inflammation, an observation that has previously been documented in obese adults [40,41]. Taken together, and similar to civilian populations, obesity may be a primary cause of metabolic dysfunction as none of the submariners in the non-obese group met the criteria for metabolic syndrome or insulin resistance.

These data combined with our previous research [29] confirm an obesity prevalence similar to the general population, however, as to whether the submarine patrols are a contributing factor is unknown. By re-examining the crewmembers immediately upon return from a patrol we learned that the time spent submerged, up to 3-months, is not the cause. In spite of 18-hour days and rotating shift work, and a reduction in sleep, the obese sailors lost ~5 and 11% of their body and fat mass, respectively, while maintaining their fat-free mass. These changes can be attributed to a reduction in energy intake, since reported physical activity did not change during the patrol and was likely sufficient to maintain protein stores. In addition, the percentage of obese submariners that had metabolic syndrome decreased by 12% and insulin resistance by 9%. Furthermore, there was an overall improvement in serum lipids and a reduction in IP-10 and MCP-1. IP-10 disturbs the migration of smooth muscle cells and endothelial permeability, and MCP-1 stimulates the migration of monocytes and other leukocytes to the endothelium increasing vascular inflammation [42]. LDL-C, inflammation, and chemotaxis of macrophages and lymphocytes are considered requisite components for atheroma formation in the vasculature wall [43]. Although modest, the reduction in LDL-C, IP-10 and MCP-1 suggest a favorable milieu for vascular function potentially reducing the development/progression of atherosclerosis, which has been reported in ~11% of servicemembers with no cardiovascular risk factors and as high as 50% in those with dyslipidemia [44].

The overall mean reduction in weight and fat mass observed herein was, however, insufficient to counteract the full complement of metabolic dysfunction as 70% of the obese group still had a BF% exceeding 25%. And from this group, in spite of a significant decrease, serum leptin remained two-fold greater than the non-obese submariners. Even so, this reduction and an overall trend for an increase in the gastrointestinal hunger hormone ghrelin would favor feeding in order to replete energy, a survival mechanism that can lead to weight regain [45,46]. These physiological changes would not favor continued weight loss unless dietary restriction is continued and physical activity is increased during the in port period, behaviors necessary for the avoidance of T2DM and CVD.

To our knowledge this is the first study examining submariner cardiometabolic health during a routine ballistic missile submarine patrol. Due to the occupational demands placed on submariners during deployments, this research is not without limitations. First, while the observed improvements in body composition and metabolic health are intriguing, we acknowledge that only a cohort of submariners of one submarine crew within the U.S. Navy were studied, and the results may not be generalizable to all submariners. Second, no direct hormonal measurements of circadian entrainment or misalignment were obtained; however, circadian misalignment has previously been documented in submariners who followed a similar work-rest schedule as in the present study [7]. Since the mean number of hours spent sleeping each day decreased during the patrol, it would appear that the submariner’s circadian rhythms were misaligned. Third, the use of accelerometers, indirect calorimetry or doubly-labeled water would have been preferable for a more accurate assessment of energy expenditure.

## 5. Conclusions

The results from this research are in agreement with previous data documenting a high prevalence of obesity in submariners that is similar to the U.S. general population. Within the obese volunteers, 30% met the criteria for metabolic syndrome, increasing their risk of developing coronary heart disease, CVD and T2DM by ~2.5 to 7-fold [47]. The observed increases in GRO, MDC and RANTES observed in the obese submariners likely indicate the presence of inflammation. However, in spite of confined space, shift work and reduction in sleep, a 3-month submarine patrol does not worsen obesity and cardiometabolic health, as evidenced by modest improvements in body composition, serum lipids, IP-10 and MCP-1. These positive changes, most notably the 5% mean reduction in body mass, were, nonetheless, insufficient to completely reverse metabolic dysregulation. In fact, a reduced leptin and overall increase in ghrelin favors energy repletion, thus weight regain may occur during in port periods. Although only a cohort of a submarine crew was examined before and after a single patrol, the increased prevalence of obesity in the U.S. military as a whole strengthens the importance of the findings presented herein. Future interventional studies should be conducted in order to not only ensure that the current U.S. military force is healthy while serving, but also following active duty separation.

## Disclaimer

“The opinions or assertions contained herein are the private views of the authors and are not to be construed as official or as reflecting the views of the Department of the Navy, Department of the Army, the Department of Defense, or the U.S. Government. Any citations of commercial organizations and trade names in this report do not constitute an official Department of the Navy, Department of the Army, or Department of Defense endorsement of approval of the products or services of these organizations. I am a military service member (or employee of the U.S. Government). This work was prepared as part of the authors official duties. Title 17 U.S.C. §105 provides that “Copyright protection under this title is not available for any work of the United States Government.” Title 17 U.S.C. §101 defines a U.S. Government work as a work prepared by a military service member or employee of the U.S. Government as part of that person’s official duties.”
